# Cardiac cytoarchitecture — why the “hardware” is important for heart function!^☆^

**DOI:** 10.1016/j.bbamcr.2015.11.006

**Published:** 2016-07

**Authors:** Elisabeth Ehler

**Affiliations:** BHF Centre of Research Excellence at King's College London, Cardiovascular Division and Randall Division of Cell and Molecular Biophysics, London, UK

**Keywords:** Intercalated disc, Myofibril, M-band, Formin, Dilated cardiomyopathy

## Abstract

Cells that constitute fully differentiated tissues are characterised by an architecture that makes them perfectly suited for the job they have to do. This is especially obvious for cardiomyocytes, which have an extremely regular shape and display a paracrystalline arrangement of their cytoplasmic components. This article will focus on the two major cytoskeletal multiprotein complexes that are found in cardiomyocytes, the myofibrils, which are responsible for contraction and the intercalated disc, which mediates mechanical and electrochemical contact between individual cardiomyocytes.

Recent studies have revealed that these two sites are also crucial in sensing excessive mechanical strain. Signalling processes will be triggered that## lead to changes in gene expression and eventually lead to an altered cardiac cytoarchitecture in the diseased heart, which results in a compromised function. Thus, understanding these changes and the signals that lead to them is crucial to design treatment strategies that can attenuate these processes. This article is part of a Special Issue entitled: Cardiomyocyte Biology: Integration of Developmental and Environmental Cues in the Heart edited by Marcus Schaub and Hughes Abriel.

## Highlights

• General description of cardiac cytoarchitecture during development and disease with an emphasis on cytoskeletal proteins
• Cardiac cytoarchitecture and signalling in cardiomyopathy

**Fig. 1:**
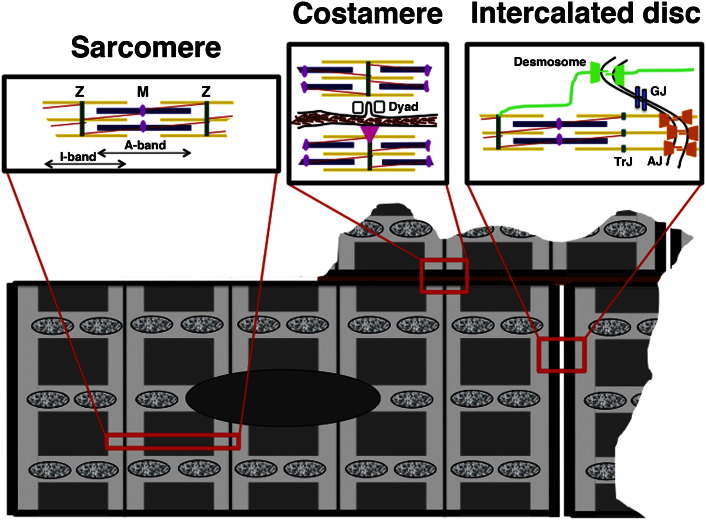
Schematic drawing of a cardiomyocyte. The basic unit of the myofibril, the sarcomere, the contacts between the cardiomyocyte and the extracellular matrix (ECM) and the contacts between two neighbouring cardiomyocytes; the intercalated discs are shown in insets above the cell. Mitochondria are represented as mottled ovals. Sarcomere: Z-discs, the lateral borders of a sarcomere, are depicted in dark green and anchor the thin filaments (actin and associated proteins) shown in yellow. The thick filaments (myosin and associated proteins) are shown in dark blue and are linked with the elastic filaments (titin; shown in red) at the M-band in the middle of the sarcomere (shown in purple). Costamere: The myofibrils are linked to the plasma membrane at Z-disc level via the costameres (shown with a pink triangle in the lower cell). Also at Z-disc level are specific membrane invaginations, the T-tubules, which associate with flanking SR to the Dyad (shown in the upper cell). ECM is depicted in brown. Intercalated disc: Desmosomes (shown in green) and adherens junctions (AJ, shown in orange) link neighbouring cardiomyocytes mechanically while gap junctions (GJ, shown in light blue) provide ion channels for intercellular communication. Desmosomes link up to the intermediate filament cytoskeleton (composed of desmin), while adherens junctions anchor actin filaments (the myofibrils). The border of the last sarcomere before the plasma membrane is defined as the transitional junction (TrJ).

Cells that make up fully differentiated tissues are characterised by a shape and subcellular organisation that enables them to carry out whichever task they have to perform. In case of the cells that make up the contractile tissue of the heart, the cardiomyocytes, this high degree of organisation is already apparent at the light microscopy level by a more or less rectangular shape of the isolated cells and a cross-striated appearance in phase contrast, which is due to the paracrystalline arrangement of the contractile elements, the myofibrils. This amazing degree of organisation is not only seen at the level of the cardiomyocyte itself, but also how they are combined to a tissue [1]. On longitudinal sections through healthy heart tissue, cell–cell contacts are exclusively found at the narrow ends of the bipolar cardiomyocytes, hence their name intercalated discs, while cell–matrix contacts ensheath the cells laterally (Fig. 1). This organisation is crucial for the directed propagation of electrical signals through serial strands of cardiomyocytes. In disease, this strict order is compromised, ultimately leading to arrhythmias that can even cause sudden cardiac death. This high degree of organisation is also a major obstacle for any attempts to regenerate damaged heart tissue for example after a myocardial infarction. No matter, which replacement strategy is considered (re-activation of cell division of endogenous cardiomyocytes; injection of contractile cells; transplantation of cardiomyocytes in scaffolds or in patches), if there is no directed electrochemical contact and controlled mechanical integration with the host tissue, life-threatening arrhythmias are the likely consequence [2]. This review will focus on cytoskeletal multiprotein complexes, which are at the basis of cardiac cytoarchitecture, the myofibrils and the intercalated discs (Fig. 1).

## 1 The myofibrils

The myofibrils make up the contractile machinery, in which the contractile partners actin and myosin are arranged to interdigitating thin and thick filaments in a way that ensures maximal force output. The basic unit of a myofibril is called the sarcomere and is defined as the region between two Z-discs. The actin crosslinker protein α-actinin is a classical marker for the Z-disc; however the Z-disc harbours a plethora of other cytoskeletal and signalling proteins [3]. The Z-discs anchor the thin filaments, which are composed of actin, tropomyosin and the troponin complex. Tropomyosin and the troponin complex are crucial for regulation of contraction at the thin filament level, which is triggered by calcium. The thin filaments are capped at their barbed ends in the Z-disc by CapZ [4] and at their pointed ends, which stretch towards the middle of the sarcomere by tropomodulin [5]; for recent reviews of thin filament length control see Refs. [6], [7]. The thick filaments are composed of dimers of myosins (a myosin consists of one myosin heavy and two myosin light chains), which are arranged to bipolar filaments with the myosin tails making up the central region of the sarcomere and the region of the heads interdigitating with the thin filaments. MyBP-C (Myosin binding protein C; C-protein) is associated with a subset of the myosin heads and contributes to the control of contraction at the thick filament level [8]. The third filament system is called the elastic filaments and is made up of titin (previously called connectin [9]). One titin molecule stretches from the Z-disc, where its N-terminus is anchored to the M-band, where its C-terminus overlaps with titin stretching from the other half of the sarcomere. Due to its position in the sarcomere and the observation that cells lacking titin are unable to assemble sarcomeres [10], titin has been attributed with an absolutely crucial role during myofibrillogenesis [11]. The M-band is the molecular structure that links elastic and thick filaments and is necessary to keep thick filaments centred in the sarcomere. Its main marker protein is myomesin, which gets localised together when the first organised A-bands are seen [12]. Thus M-bands are defined molecularly by the presence of myomesin rather than an electron dense appearance in the electron microscope, which is only attained postnatally. Depending on muscle type and developmental stage additional M-band proteins such as M-protein, the splice variant of myomesin, EH-myomesin or myomesin-3 are added to myomesin in the M-band [13].

## 2 Protein turnover in the sarcomere

The somewhat static arrangement of the sarcomere that is suggested by the regular appearance of sarcomeres in electron micrographs, is probably an oversimplification. Changed physiological demands lead to adaptations for example by switching the expression levels of myosin heavy chain isoforms [14]. In addition, observations on the protein turnover of thin filament components have indicated a half-life between 3 and 10 days in the rat heart [15]. This would suggest that every 1 to 2 weeks, the molecular makeup of a sarcomere is exchanged. Since these measurements were carried out at the whole heart level, it is unclear, whether different heart regions display a different dynamics in their protein turnover and how much this is affected for example by the expression of mutant and/or aggregating proteins. As far as actin turnover is concerned, it was revealed in recent studies that proteins, which are well known to affect actin polymerisation such as N-WASP, leiomodin or the formin family member FHOD3 are expressed in striated muscle and are located in the sarcomere either at the barbed (N-WASP, FHOD3) or the pointed ends (leiomodin) of the thin filaments [16], [17], [18]. Their position in the sarcomere and extent of expression is dependent on the developmental stage or on physiological stress [19], [20], [21].

## 3 The intercalated disc

The intercalated discs ensure electrochemical and mechanical connection between neighbouring cardiomyocytes and are situated at the end of a cardiomyocyte [22]. According to the classical view they are composed of three different types of cell–cell contacts: adherens junctions, desmosomes and gap junctions. Adherens junctions anchor the actin filaments (the myofibrils) and are composed of N-cadherin as a transmembrane component that provides the link to the neighbouring cell and a cytoplasmic plaque that contains amongst others α-catenin and β-catenin and connects to the actin filaments. The intercellular link in desmosomes is provided by the desmosomal cadherins, desmoglein and desmocollin, while the cytoplasmic plaque consists of desmoplakin, plakophilin and plakoglobin, which link to the intermediate filament cytoskeleton, which is made up by desmin in muscle cells. Gap junctions consist of connexin-43 in ventricular cardiomyocytes and provide ion channels for intercellular communication (Fig. 1). This simple classification has been challenged recently by observations that find classical desmosomal proteins such as desmoplakin, plakophilin and plakoglobin also in adherens junctions by immunoelectron microscopy. Therefore the terminology of “area composita” was introduced to describe plaque-bearing cell–cell contacts at the intercalated disc [23].

## 4 Lateral plasma membrane organisation

While the myofibrils are anchored at their ends by the adherens junctions, their sides are also attached to the plasma membrane at regular intervals at the costameres, which are situated at the Z-disc level [24]. A classical marker for costameres is vinculin [25] and they provide via integrins the link to the extracellular matrix that covers cardiomyocytes laterally (shown in brown in Fig. 1). In addition, there are membrane invaginations at Z-disc level in cardiomyocytes, the T-tubules, which are flanked by sarcoplasmic reticulum (SR) and make up the dyad structure, which is necessary for excitation contraction coupling [26]. Cytoplasmic actins together with a specific tropomodulin isoform play an important role in their organisation [27], together with the protein Bin1, which is required for shaping T-tubules [28] and the protein junctophilin-2, which defines the distance between the plasma membrane and the membrane of the SR [29]. Within the SR, proteins such as triadin and to a lesser extent junctin [30], [31] were shown to be important for the organisation of diads in mice and recently mutations in triadin were shown to be a possible cause of sudden cardiac death in humans [32].

## 5 Establishment of cytoarchitecture during development

The regular cytoarchitecture described above is only attained after birth in the heart. While myofibrils are assembled and contraction is observed from embryonic day 9 onwards in mouse hearts, the myofibrils are still very thin and not well aligned laterally [33]. The Z-disc associated protein telethonin (also known as T-cap), which links two N-termini of titin in a sandwich like complex [34], a structure which is required for maximal stability of sarcomeres [35], is only expressed in a subset of neonatal rat cardiomyocytes and also the switch in M-band composition from a more immature state marked by the presence of the more elastic EH-myomesin isoform to the mature state, which is characterised by the incorporation of M-protein and the appearance of M1-lines at the EM level, is only achieved postnatally [36]. The sparser arrangement of myofibrils, less lateral crosslinking via desmin and the lack of maturity of the sarcomeres makes them more pliable for disassembly, which is a prerequisite for successful cell division of cardiomyocytes [37]. In the embryonic heart, the cell–cell contacts are distributed over the entire surface of the cardiomyocytes and become restricted concomitant with a cellular elongation and better myofibril alignment only around birth [33]. Also the organisation of the plasma membrane and the sarcoplasmic reticulum to dyads is only achieved after birth [26].

In the first postnatal week a switch from hyperplastic (i.e. growth via cell division) to hypertrophic (i.e. growth via increase in cell size) takes place in the heart [38] and the ensuing increase in cellular girth and increased organisation of their cytoarchitecture contributes to making cell division a more unfavourable process. Elegant studies by Porrello and colleagues have shown that neonatal mouse hearts still possess the ability to regenerate by reactivation of cell division in cardiomyocytes and that this ability gets lost within the first week after birth [39]. Recent studies have correlated a regenerative capacity with a specific transcriptional signature, which resembles a more immature phenotype [40]. It was postulated that the trigger for the switch may be the oxygen-rich environment after birth and a residual population of smaller cardiomyocytes with hypoxic characteristics and increased proliferative potential was identified [41]. Zebrafish cardiomyocytes retain their proliferative capacity also at adult stages [42], but they are long and slender and do not have dyads [43]. Cardiomyocyte division in the adult would require the disassembly of the myofibrils, the disassembly of their sophisticated membrane organisation and the disintegration of the separated cell–cell and cell–matrix contact arrangement and is clearly not trivial to achieve. This is probably the explanation, while a lot of attempts to induce cell division in cardiomyocytes by the forced expression of cell cycle regulating factors have met with limited success, but also has the positive side effect that cancerous growth of cardiomyocytes is extremely rare [44]. There are reports of cardiomyocyte turnover to a varying degree in human heart [45], [46] and it is also well established that there are several populations of stem cell-like properties in the mammalian heart [47], [48], [49], [50]. However, under normal circumstances these activities are insufficient to deal with heart tissue loss for example following a myocardial infarction. Challenges that lead to pathological growth of cardiomyocytes result in additional DNA synthesis and even the re-expression of cytokinesis markers in cardiomyocytes [51], but significant cell division activity can usually not be observed. Recently it was demonstrated that a reluctance to divide in adult mammalian cardiomyocytes can also be explained by a loss of centrosome integrity immediately after birth, which does not happen in zebrafish cardiomyocytes [52]. Taken together all these sophistications that characterise postnatal mammalian cardiomyocytes contribute to their inability to divide and make it a formidable task to switch them back to a more immature, dividing state. It is unlikely that a single magic molecular bullet will be sufficient to achieve this, but broader interference at an epigenetic level will probably be required.

## 6 Why does cardiac cytoarchitecture matter?

As described above the high degree of organisation at the tissue and cellular level in the heart is needed for maximal functional output with every heart beat. A first insight that even a point mutation in one of the cytoskeletal proteins that make up this cytoarchitecture can have grave consequences, was the discovery that a single amino acid change in the myosin heavy chain molecule can be correlated with hereditary cardiomyopathy, in this case familial hypertrophic cardiomyopathy [53]. It is now well established that mutations in proteins that make up the myofibrils or the intercalated discs lead to hereditary cardiomyopathies [54], [55], [56], [57]. The majority of point or frameshift mutations in myofibrillar proteins lead to hypertrophic cardiomyopathy [54], while truncations in titin were recently shown to account for a fifth of hereditary dilated cardiomyopathy [58]. Mutations in desmosomal proteins lead to arrhythmogenic right ventricular cardiomyopathy and to sudden cardiac death, potentially via an effect on connexin-43 in gap junctions [59], [60]. The list of cardiomyopathies clearly associated with mutations will expand dramatically due to next generation sequencing in the next years; however, it is interesting to bear in mind that despite mutations in distinct proteins, the general histology observed in hypertrophic cardiomyopathy (HCM) and dilated cardiomyopathy (DCM) seems to be mutation independent.

**Fig. 2:**
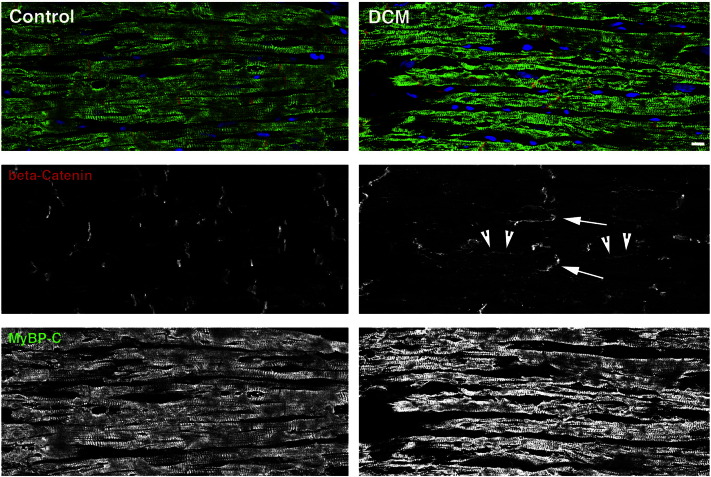
Confocal micrographs of human heart tissue immunostained for the intercalated discs and the myofibrils. Longitudinal cryosections of human heart tissue from a control individual (male, 23 years) and a dilated cardiomyopathy (DCM) patient (male, 31 years) were stained with mouse monoclonal antibodies against β-catenin (red signal and middle row) and rabbit polyclonal antibodies against MyBP-C (green signal and bottom row). Nuclei were stained with DAPI and are shown in blue. While overall tissue arrangement and myofibril alignment are relatively unaffected in DCM, an increased signal for β-catenin compared to control can be observed at the intercalated discs (arrows) and also laterally (arrowheads) in DCM. Scale bar is 10 μm.

HCM leads to myocyte and myofibril disarray, which is clearly apparent even at the histological level, while heart tissue from DCM patients can look relatively unharmed apart from obvious areas of myocyte dropout and fibrosis. The changes in cardiac cytoarchitecture that are seen in DCM are subtle and mainly affect the intercalated disc. The plasma membrane between neighbouring cardiomyocytes gets more convoluted, resembling cell–cell contacts from aged hearts [61]. This is accompanied by a more disorganised insertion of novel sarcomeres at the intercalated disc region in DCM [62]. In addition, an increase in actin anchoring components and also of F-actin is detected [63]. This may in part be due to the increased presence of a formin, FHOD1, at the intercalated discs of DCM hearts [64]. However, not all formins are upregulated in their expression in DCM, since FHOD3, which plays a role in myofibril maintenance in the embryo [65] and in cultured cardiomyocytes [17] was seen to be downregulated in human cardiomyopathy, potentially contributing to myofibril loss. Another change that is seen in DCM cardiomyocytes is the switch to more elastic isoforms of titin and myomesin in the myofibrils [66], [67]. Cardiac cytoarchitecture is also affected as far as integration to a tissue is concerned, since for example the strict segregation of cell–cell and cell–matrix contacts on the surface is lost and for example adherens junction proteins such as β-catenin can also be detected at the sides of cardiomyocytes, especially in HCM [68] but also occasionally in DCM (Fig. 2). The expression of connexin-43 is dowregulated in murine DCM and thus there are fewer gap junctions available to mediate intercellular communications [63]. This phenotype is less penetrant in human DCM samples [69], but a mislocalisation of connexins was also described in HCM and following myocardial infarction [70]. Also the lateral membrane organisation gets lost in cardiomyopathy, with T-tubules and dyads becoming less organised and costameres as visualised by vinculin losing their regular striated appearance [28], [63], [71], [72], [73].

It has been shown that mutations in myosin, tropomyosin and titin can result in different phenotypes such as HCM, DCM and even arrhythmogenic right ventricular cardiomyopathy (ARVC) and the correlation between a mutation in a particular protein and the disease phenotype is obviously not as clear-cut as initially proposed [74]. The histological and molecular analysis of human material is usually restricted to end-stage hearts upon transplantation or death. It is clear that during the aetiology of cardiomyopathy, a more fluid interchange between phenotypes may be seen that can only be followed in animal models for cardiomyopathy. A particular mutation will affect protein–protein interactions, activity, subcellular targeting or stability of a particular molecule, but the phenotype is caused by the impairment at the cellular level and any compensatory mechanisms (e.g. posttranscriptional control of expression; different protein turnover) that were activated.

## 7 What leads to an altered cardiac cytoarchitecture in cardiomyopathy?

**Fig. 3:**
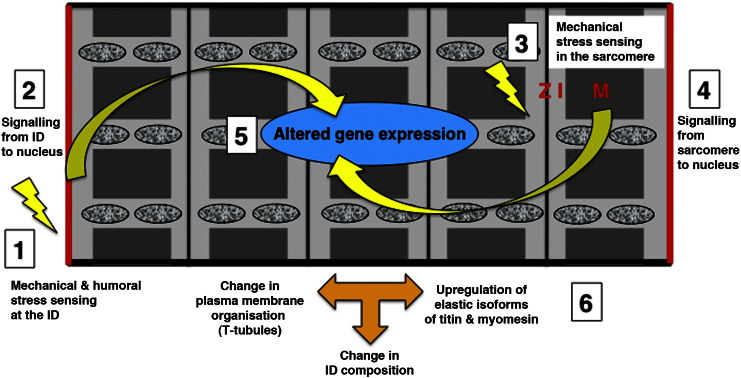
Schematic representation of signalling events in dilated cardiomyopathy (DCM). The two major sensors of mechanical stress are the intercalated discs (1) and the Z-disc, I-band and M-band region of the sarcomeres (3). Several signalling cascades (2, 4) lead to altered gene expression (5), which eventually results in a different composition of the intercalated disc (upregulation of actin-anchoring proteins) and the sarcomeres (re-expression of more elastic embryonic isoforms of titin and myomesin) and changes in overall cytoarchitecture such as an altered shape, loss of strict size control and loss of high degree of plasma membrane organisation (6). Mitochondria are represented as mottled ovals.

The myofibrils and the intercalated discs are not only essential for the function of the heart tissue, but are also centres for mechanosensing and signalling (Fig. 3) [75]. For example the Z-disc harbours not only structural proteins such as α-actinin but also kinases (PKCepsilon) and phosphatases (calcineurin) that are well known to be involved in signalling processes in cardiomyocytes [3]. Some cytoskeletal proteins also contain PDZ domains, which act to establish proximity to plasma membranes since lipids are needed for the activation of small GTPases and kinases such as PKCα [76], [77]. Cytoskeletal proteins often also contain SH3 domains or LIM domains, which provide hubs for protein–protein interaction [78], [79]. For example FHL1, which is a LIM domain containing protein that associates with titin's I-band region is involved in mediating signalling via ERK to the nucleus to trigger a hypertrophic response [80], [81]. Another mechansosensitive protein in the I-band is CARP, which can trigger signalling, but can also translocate to the nucleus itself [82]. MLP (muscle LIM protein) can also switch its localisation from the cytoplasm to the nucleus, depending on the mechanical stretch that a cardiomyocyte experiences [83]. While the Z-discs deal mainly with transversal forces during active contraction and are hardly affected in their longitudinal arrangement, the M-bands (together with titin) were shown to be crucial for dealing with longitudinal forces and with centering the A-bands following contraction [84]. Interestingly, titin harbours a kinase domain in its M-band region, whose active site becomes accessible upon mechanical stretch [85]. Whether this results in actual kinase activity or opens up protein–protein interaction sites that subsequently trigger a signalling cascade towards the nucleus is under debate at the moment [86], [87], but the involvement of MURF2 (a muscle specific E3 ubiquitin ligase), p62 (known to be involved in autophagy) and SRF (a transcription factor with a well-established role in the heart) suggests a fundamental role at different levels.

The redistribution of signalling components that accompanies cardiomyopathy also contributes to changes in the downstream readout. For example, the loss of lateral membrane organisation in failing hearts leads to β2-adrenergic receptors no longer being concentrated at T-tubules but being distributed more homogenously over the cell surface, with a concomitant loss of tightly localised downstream signalling [73]. Re-organisation of the intercalated discs in DCM is accompanied by a concentration of PKCα there [88], which is known to contribute to pathological signalling [89]. Recently it was shown that cardiomyocytes differentiated from iPS cells from patients with titin truncations show an impaired signalling via ERK, p38 and AKT pathways [90], again linking malfunctional sarcomeres with altered signalling.

Exactly how the alterations in signalling that are seen in cardiomyopathy lead to the observed changes in cytoarchitecture is not well understood. It was shown that changes that affect alternative splicing can be correlated with cardiomyopathy [91]. Mutation in the splicing associated RNA-binding protein Rbm20 are found in DCM patients and could result in alternative splicing of titin and myomesin [92]. Factors that control transcription at the epigenetic level such as miRNAs and lncRNAs are exciting candidates to cause the more pleiotropic changes that are seen in cardiomyopathy [93], [94], [95] and may turn out to be an elegant tool to interfere with maladaptive signalling processes that result in altered cardiac cytoarchitecture.
